# Antibacterial activity of mangrove-derived *Streptomyces pseudogriseolus* NELs-50 against methicillin-resistant *Staphylococcus epidermidis*

**DOI:** 10.4102/ajlm.v15i1.3214

**Published:** 2026-10-20

**Authors:** Yutian Qin, Wenqing Wang, Mamy Jayne Nelly Rajaofera

**Affiliations:** 1 National Health Commission Key Laboratory of Tropical Disease Control, School of Life Sciences and Medical Technology, Hainan Medical University, Haikou, China

**Keywords:** MRSE, *Streptomyces pseudogriseolus*, mangrove microorganisms, antibacterial activity, bioactive compounds, GC-MS analysis

## Abstract

**Background:**

Methicillin-resistant *Staphylococcus epidermidis* (MRSE) represents a growing healthcare threat, necessitating novel antimicrobial agent development. Mangrove-derived *Streptomyces* species offer promising potential as sources of bioactive compounds against drug-resistant pathogens.

**Objective:**

To isolate and characterise a mangrove-derived *Streptomyces* isolate with anti-MRSE activity and identify its bioactive metabolites.

**Methods:**

Mangrove soil samples were screened for anti-MRSE activity. Isolate NELs-50 was identified through morphological, biochemical, and 16S rRNA gene sequencing. Fermentation parameters were optimised, bioactive metabolites extracted with organic solvents, and characterised using gas chromatography-mass spectrometry (GC-MS). Minimum inhibitory concentration (MIC) and stability assessments were conducted.

**Results:**

NELs-50 was identified as *Streptomyces pseudogriseolus* (99% sequence similarity) with potent anti-MRSE activity (28.67 mm inhibition zone). Culture conditions were optimised for maximum antimicrobial production. Ethyl acetate extract showed highest activity (28.83 mm). The extract retained 41% activity at 121 °C, tolerated pH 2–12, and resisted enzymatic degradation. Gas chromatography-mass spectrometry analysis identified nine compounds, with major components being pyrrolo[1,2-a]pyrazine derivatives (100% peak area), and 2,4-Di-tert-butylphenol (12.64%). Minimum inhibitory concentration against MRSE was 125 µg/mL.

**Conclusion:**

*S. pseudogriseolus* NELs-50 shows promising anti-MRSE activity and produces putative bioactive metabolites with broad pH tolerance but reduced activity at elevated temperatures, supporting its potential as a source of antimicrobial lead compounds.

**What this study adds:**

This study reports the isolation and characterisation of *S. pseudogriseolus* NELs-50 from Hainan mangroves. The ethyl acetate extract showed broad pH tolerance but was heat-sensitive at elevated temperatures, retaining only 41% activity after treatment at 121 °C. Gas chromatography-mass spectrometry analysis identified nine putative metabolites, suggesting that NELs-50 may serve as a promising source of antimicrobial lead compounds against multidrug-resistant pathogens.

## Introduction

Methicillin-resistant *Staphylococcus epidermidis* (MRSE) has emerged as an important nosocomial pathogen in healthcare environments worldwide.^1^ This coagulase-negative *Staphylococcus* is particularly associated with infections involving medical devices and biofilm formation.^2^ The resistance profile of MRSE, including methicillin resistance and potential resistance to other antimicrobial agents, presents therapeutic challenges for clinicians.^3^ Multidrug-resistant *S. epidermidis* is responsible for difficult-to-treat infections and hospital-acquired infections, with more than 80% of nosocomial isolates demonstrating methicillin resistance.^4^ These factors highlight the ongoing need for research into novel antimicrobial agents and treatment strategies.

*Streptomyces* species have historically served as nature’s most prolific pharmaceutical factory, producing over 70% of naturally derived antimicrobial agents currently in clinical use, including streptomycin, tetracycline, erythromycin, and chloramphenicol.^5,6,7^ These soil-dwelling actinomycetes possess extraordinarily complex secondary metabolite biosynthetic pathways, often encoding 20–30 distinct biosynthetic gene clusters per genome, representing a vast reservoir of chemical diversity with untapped therapeutic potential.^2,8,9^ While terrestrial *Streptomyces* strains have been extensively studied and exploited over the past seven decades,^10,11^ marine and mangrove-associated strains represent an underexplored frontier in natural product discovery.^12^ Mangrove ecosystems, characterised by extreme environmental conditions including high salinity, temperature fluctuations, nutrient limitation, and unique chemical gradients, exert selective pressures that drive the evolution of novel biosynthetic capabilities.^13,14^ These distinctive ecological niches harbour *Streptomyces* populations with unique phylogenetic profiles and metabolic capacities, often producing structurally unprecedented compounds with potent biological activities against drug-resistant pathogens.^8,10,15^

Despite growing interest in marine-derived antimicrobials, the anti-MRSE potential of *Streptomyces* from Hainan mangrove environments has received limited investigation. Hainan’s mangrove ecosystems constitute a biodiversity hotspot containing numerous uncharacterised microbial taxa with potential pharmaceutical applications.^16,17^ Previous research efforts have primarily focused on broad antimicrobial screening rather than targeted anti-MRSE evaluation, and comprehensive chemical profiling of bioactive compounds from these ecosystems remains incomplete and unexplored.^18,19,20,21^ The present study implements an integrated experimental approach encompassing isolation, screening, molecular taxonomic identification, culture condition optimisation, and detailed chemical characterisation using GC-MS. This approach systematically evaluates anti-MRSE activity in mangrove-derived *Streptomyces* isolates from Hainan province.

## Materials and methods

### Ethical considerations

This study involved only bacterial isolates *S. epidermidis* American Type Culture Collection (ATCC) 12228, a laboratory stock collection and *Streptomyces* sp. NELs-50 isolated from mangrove soil in Dongfang City, Hainan, China. All laboratory procedures were conducted in accordance with standard microbiological safety protocols and institutional biosafety guidelines. No human subjects, human-derived materials, or animal subjects in this research. Therefore, ethical clearance was not required. A formal ethical waiver statement from the School of Life Sciences and Medical Technology, Hainan Medical University, Haikou, Hainan, 571199, China, was obtained, confirming that this study falls outside the scope requiring institutional ethics committee approval.

### Test organism

The MRSE strain used in this study was obtained from the laboratory culture collection and maintained at –80 °C in glycerol stocks. The strain was routinely sub-cultured on lysogeny broth (LB) agar plates and incubated at 37 °C for 24 h to obtain fresh cultures for each experiment.

### Soil sampling and microbial isolation

This project commenced on 01 September 2024 and concluded on 30 June 2025. Mangrove soil samples (~500 g) were randomly collected from coastal areas in Dongfang city, Hainan province, China (19.09515°N, 108.181°E), at 0 cm – 30 cm depth, air-dried at room temperature, and stored in sealed polyethylene bags. A 1 g soil sample was suspended in 10 mL sterile distilled water, incubated at 37 °C with shaking (180 rpm, 15 min), then allowed to settle for 5 min before preparing ten-fold serial dilutions. Aliquots of 100 µL were plated onto Petri dishes containing International Streptomyces Project (ISP)-1 medium and incubated at 37 °C for 5 days, after which isolates showing antibacterial activity were purified and selected for further analysis.

### Strain identification

The taxonomic identification of the isolate was carried out following the experimental workflow detailed in a previous investigation,^22^ which combines conventional phenotypic characterisation with molecular phylogenetic analysis. Accordingly, morphological and biochemical profiling were conducted in line with Bergey’s Manual criteria. Genomic DNA was extracted using a Tiangen kit, and the 16S rRNA gene was amplified in 25-µL reactions with the universal primers 27f/1492r. The thermal cycling conditions consisted of an initial denaturation at 95 °C for 5 min, 30 cycles of 94 °C (30 s), 50 °C (60 s), and 72 °C (60 s), and a final extension at 72 °C for 10 min. Following purification and verification by agarose gel electrophoresis, the amplicons were sequenced bidirectionally. The resulting sequences were compared against the National Center for Biotechnology Information (NCBI) GenBank database via BLASTn. For the phylogenetic reconstruction, the neighbour-joining algorithm was implemented in Molecular Evolutionary Genetics Analysis (MEGA) version 7.0, and nodal support was evaluated with 2000 bootstrap pseudo replicates.^23^

### Antibacterial activity testing

The production of antibacterial compounds during submerged fermentation was quantified via the standard agar well-diffusion technique.^24^ Briefly, the antagonistic strain was propagated in tryptic soy broth (TSB) and maintained under fermentation conditions for a total of 5 days prior to the assay. The supernatant was collected by centrifugation at 8000 × g for 20 min at 4 °C followed by filtration of the supernatant through a 0.2 µm membrane filter. Briefly, the test organism was seeded in molten agar medium precooled to 45 °C. Sterile 6-mm paper discs were independently impregnated with 10 µL of the filtered supernatant under aseptic conditions before being carefully placed onto the surface of the inoculated agar plates. The plates were incubated at 37 °C for 48 h, after which antibacterial activity was determined by measuring the diameter of the inhibition zones.

Culture conditions for antimicrobial production were optimised using Plackett-Burman design with Design-Expert software. Five independent variables (Online Supplementary Table 1) including temperature, pH, agitation speed, inoculation time, and inoculum size, were tested at high (+1) and low (−1) levels across 12 trials conducted in triplicate (Online Supplementary Table 2). Antibacterial activity against the test organism was measured by inhibition zone diameter as the response variable. Data were analysed at 5% significance level (*p* < 0.05) using the regression equation (Equation 1):


$$\begin{array}{l} \text{Y}=\beta_{0}+\beta_{1}{\text{X}}_{1}+\beta_{2}{\text{X}}_{2}+\ldots +\beta_{\text{n}}{\text{X}}_{\text{n}},\text{ where Y is the predicted response,}\\ \beta_{0}\text{ is the intercept},\;\beta_{1}{-}_{\text{n}}\text{ are variable coefficients, and }{\text{X}}_{1}{-}_{\text{n}}\text{ are coded}\\ \text{values of independent variables}. \end{array}$$ [Eqn 1]

Model adequacy was determined through ANOVA, and quality was assessed using coefficient of determination (*R*^2^) and adjusted *R*^2^.

### Extraction of antibacterial metabolites

Liquid fermentation was selected for the extraction of antibacterial metabolites. The fermentation process was carried out in 1000 mL flasks containing 500 mL of culture medium. Following 15 days of incubation at 28 °C, the fermentation broth was subjected to centrifugation at 8000 × g for 10 min at 4 °C. The resulting supernatant was then mixed with various organic solvents. These solvents were subsequently collected, concentrated using a rotary vacuum evaporator, and the residues were resuspended in 2% dimethyl sulfoxide to achieve a final concentration of 2 mg/mL. The antibacterial effect was determined against the test organism using the well diffusion method.

### Determination of minimum inhibitory concentration

Minimum inhibitory concentration (MIC) was determined according to the previous study.^25^ For MIC evaluation, a microtiter plate dilution test was employed. The test organism was cultured to an OD_600_ of 0.3. Each well of the plate received 100 µL of fresh culture medium and 50 µL of test organisms arranged in different rows. Subsequently, 50 µL of different dilutions of freshly prepared substance was added to each well. The first column of the microtiter plate served as a blank, and the plates were read using an enzyme-linked immunosorbent assay (ELISA) plate reader (Thermo Scientific) after incubation at 37 °C for 18 h (OD_600_). The MIC was determined as the lowest concentration at which there was no increase in absorbance.

### Impact of pH, temperature, and enzymes on the antibacterial activity of the extract

For heat stability, the crude extract was incubated across a temperature range of 40 °C – 121 °C for 30 min. For pH stability, the extract was incubated over a pH range of 2.0–12.0 for 30 min at 37 °C. The extract’s susceptibility to enzymatic digestion, including proteinase K, trypsin, pepsin, and amylase, was also examined. All enzymes were purchased from Sigma Aldrich and prepared as 1.0 mg/mL solutions in 50 mM phosphate buffer (pH 7.0). Equal volumes of the enzyme solution and the active extract were mixed and incubated at 37 °C for 2 h. The enzyme reaction was halted by heating the reaction mixture at 80 °C for 10 min, and residual antibacterial activity was determined. The untreated sample was used as a control.

### Gas chromatography-mass spectrometry analysis

The compounds potentially responsible for the antibacterial activity of the isolate were identified using GC-MS technique. The Agilent Technologies 6890–5973 N with a capillary column TG-5 ms phenylmethylsiloxane (30 m × 250 µm × 0.25 µm) system was employed for the analysis. The mass detector operated in split mode, and helium gas with a flow rate of 1.0 mL/min served as the carrier. The injector was set at 230 °C, and the initial oven temperature was 60 °C for 2 min, followed by a ramp of 10 °C/min to 280 °C for 8 min. Component identification was based on the comparison of retention time and mass spectra with those in the NIST98 GC-MS library and literature data.

### Statistical analysis

Statistical analysis was performed using Statistical Package for the Social Sciences software version 26 (SPSS, Chicago, Illinois, United States). All results presented in this article were an average of three independent assays and were expressed as mean ± standard deviation (s.d.). Duncan’s multiple range tests were applied when one-way revealed significant differences (*p <* 0.05). All experiments were done in replicate of three tests.

## Results

### Strain isolation and identification

NELs-50 was isolated from mangrove soil collected in a suburban region of Hainan province, China, and subsequently evaluated for antimicrobial activity against MRSE. Among the tested isolates, NELs-50 demonstrated the most potent inhibitory activity against MRSE, producing a distinct zone of inhibition measuring 28.67 ± 0.34 mm in diameter. Comprehensive morphological and biochemical characterisation of the isolate was performed, with results summarised in Table 1.

**TABLE 1:** Comprehensive analysis of partial morphological and biochemical characteristics exhibited by the isolates (01 September 2024 to 30 June 2025, Hainan, China).

Characteristic	Result	Test item	Result
Spore mass	Grey	Cellulose hydrolysis	+
Spore surface	Spiral	Casein hydrolysis	+
Substrate	dark	Tributyrin hydrolysis	+
Catalase test	+	Nitrate reduction	+
Urease test	+	H_2_S production	-
Starch hydrolysis	+	Melanin production	-
Gelatin liquefaction	+	Milk peptisation	+

Phylogenetic analysis based on 16S rRNA gene sequencing revealed that the isolate is closely related to *Streptomyces* species (Figure 1), exhibiting 99% sequence similarity to the closest match in GenBank. The 16S rRNA gene sequence has been deposited in the GenBank database under accession number OK047741 (https://www.ncbi.nlm.nih.gov/nuccore/OK047741.1/).

**FIGURE 1:**
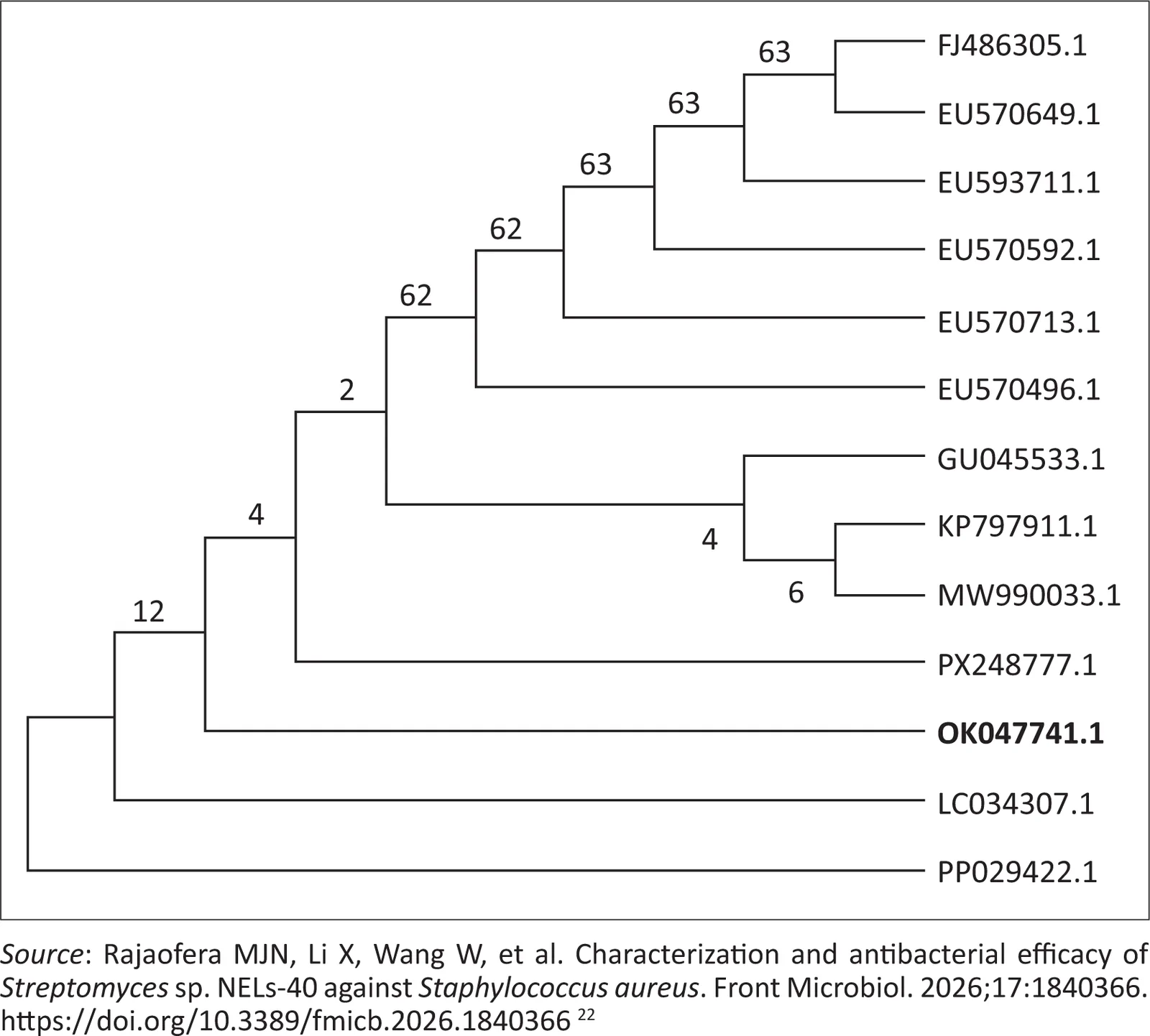
Phylogenetic relationships of strain NELs-50 inferred via the neighbour-joining algorithm using 16S rRNA sequence data, with branch support assessed by 2000 bootstrap pseudo replicates (values indicated at nodes) and a scale bar representing 0.0005 nucleotide substitutions per position (01 September 2024 to 30 June 2025, Hainan, China).

### Culture medium optimisation for antimicrobial production

Fermentation parameters were optimised to maximise antimicrobial activity against MRSE. Among five tested media (ISP-2, LB, potato dextrose broth [PDB], yeast extract peptone dextrose [YEPD], TSB), strain NELs-50 achieved maximum antimicrobial activity in TSB medium, producing a 26.41 mm inhibition zone, followed by PDB (21.30 mm), ISP-2 (10.98 mm). Negligeable activity was found in YEPD and LB (Figure 2).

**FIGURE 2:**
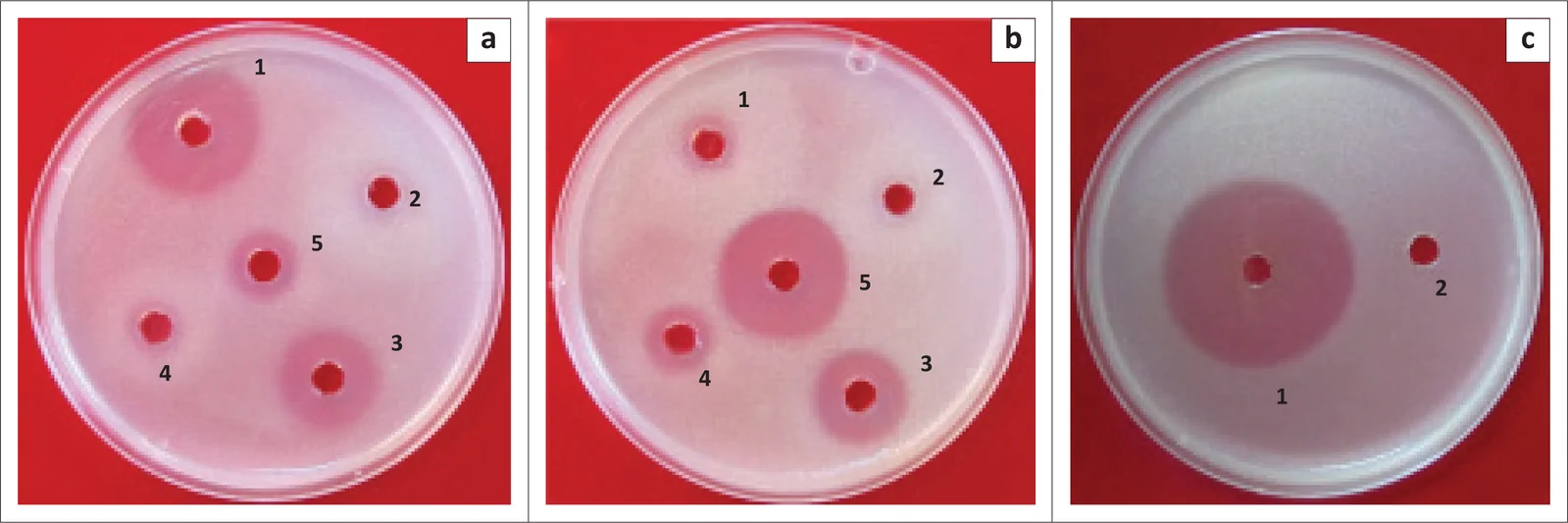
Antibacterial activity of *Streptomyces pseudogriseolus* NELs-50 against methicillin-resistant *Staphylococcus epidermidis*. (a) The isolate was cultured in different culture media and assessed for antibacterial activity (1: tryptic soy broth, 2: potato dextrose broth, 3: ISP-2, 4: lysogeny broth, 5: yeast extract peptone dextrose); (b) Crude extracts obtained using various culture solvents (1: hexane, 2: N butanol, 3: petroleum ether, 4: ethanol, 5: ethyl acetate); (c) Crude extract prepared with ethyl acetate at a concentration of 2 mg/mL (1: ethy acetate extract, 2: control which was prepared from the culture medium without organism inoculation) (01 September 2024 to 30 June 2025, Hainan, China).

### Statistical optimisation of antimicrobial metabolites

Plackett-Burman design was employed to optimise antimicrobial activity by strain NELs-50. The design matrix of 12 runs with five variables yielded inhibition zones ranging from 11.92 to 31.51 mm (Table S2). The regression model was expressed as: Y = +23.12 + 1.39A – 0.93B + 3.21C + 3.98D + 0.92E. where Y represents enzyme activity, and A–E are coded values for temperature, pH, incubation time, inoculation size, and agitation speed (Table 2).

**TABLE 2:** Analysis of the data generated by the Plackett–Burman design (01 September 2024 to 30 June 2025, Hainan, China).

Factor	Effect	Coefficient	Standard error	*p*-value	Significance
Intercept	-	23.12	0.42	0.0000	-
A	2.78	1.39	0.42	0.0104	*
B	−1.86	−0.93	0.42	0.0564	-
C	6.42	3.21	0.42	0.0003	***
D	7.96	3.98	0.42	0.0001	***
E	1.84	0.92	0.42	0.0579	-

Note: Asterisks indicate the level of statistical significance based on the *p*-value:

* , *p* < 0.05;

** , *p* < 0.01;

*** , *p* < 0.001; – not significant (*p* ≥ 0.05). The intercept is not assigned a significance code because it is not a factor in the Plackett–Burman design.

Analysis of variance (ANOVA) demonstrated high model significance (*F* = 26.89, *p* = 0.0007), with substrate concentration (D) and incubation time (C) identified as the most significant factors (*p <* 0.001). Temperature (A) and pH (B) showed significant effects (*p <* 0.05), while agitation speed (E) was not significant (*p* = 0.4067). The model showed excellent reliability with *R*^2^ = 0.9235, adjusted *R*^2^ = 0.9217, predicted *R*^2^ = 0.8345, and coefficient of variation = 6.27% (Table 3). The validation study confirmed the importance of substrate concentration and incubation time as the primary factors affecting antimicrobial activity, while demonstrating good model predictability typical of well-controlled bioprocess optimisation studies.

**TABLE 3:** Results of the analysis of variance for the regression equation (01 September 2024 to 30 June 2025, Hainan, China).

Source	Sum of square	*df*	Mean square	*F* value	*p* (prob > *F*)	Significance
Model	282.65	5	56.53	26.89	0.0007	Significant
Residual	12.62	6	2.10	-	-	-
A	23.19	1	23.19	11.03	0.0104	-
B	10.34	1	10.37	4.93	0.0564	-
C	123.16	1	123.76	58.86	0.0003	-
D	190.24	1	190.24	90.50	0.0001	-
E	10.17	1	10.17	4.84	0.0578	-
Cor Total	295.27	11	-	-	-	-

Note: Standard deviation = 1.45; Mean = 23.12; Coefficient of determination *R*^2^ = 0.9235; Adjusted coefficient of determination *R*^2^ = 0.9217; Predicted coefficient of determination *R*^2^ = 0.8345; Coefficient of variation = 6.27%; Adequate precision = 16.408.

*df*, degrees of freedom.

### Antimicrobial activity of crude extract

The fermentation process was conducted for 15 days at 28 °C using TSB medium. Following the incubation period, the culture supernatant was obtained by centrifugation at 8000 × g for 15 min. The supernatant was extracted twice with different organic solvents including ethyl acetate, petroleum ether, n-hexane, and n-butanol. The organic layers were then evaporated to dryness under reduced pressure using a rotary evaporator, resulting in crude extracts. The residual materials were dissolved in dimethyl sulfoxide (DMSO) and filtered. The extracts were subsequently tested for their antimicrobial activities. Notably, among these solvents, ethyl acetate extract exhibited the highest activity with an inhibition zone of 28.83 mm (Figure 2).

### Stability of the antimicrobial extract

The ethyl acetate extract of NELs-50 demonstrated temperature-dependent stability when treated at various temperatures for 30 min. Maximum antimicrobial activity was observed in the control with an inhibition zone diameter of 20.8 ± 0.1 mm, which remained relatively stable at 40 °C (19.5 ± 0.3 mm) and 50 °C (17.2 ± 0.2 mm) before declining significantly at higher temperatures to 13.9 ± 0.1 mm at 70 °C, 9.2 ± 0.1 mm at 100 °C, and 8.5 ± 0.1 mm at 121 °C, representing a 59% activity loss compared to the control (Figure 3). pH stability studies revealed optimal activity under control conditions (20.8 ± 0.2 mm) and at neutral to slightly alkaline pH (pH 7: 19.8 ± 0.2 mm; pH 8: 18.9 ± 0.8 mm), while acidic conditions (pH 2: 13.5 ± 0.2 mm; pH 4: 13.8 ± 0.2 mm) and strongly alkaline conditions (pH 10: 12.5 ± 0.2 mm; pH 12: 7.8 ± 0.2 mm) significantly reduced antimicrobial efficacy (Figure 3). These findings indicate the extract is thermolabile and exhibits pH-dependent stability with optimal performance under neutral to mildly alkaline conditions. Additionally, the extract demonstrated resistance to amylase, with loss in activity observed in trypsin, pepsin, and proteinase K after 2 h of enzyme treatment (Figure 3), indicating that the antimicrobial substances are slightly sensitive to these enzymes.

**FIGURE 3:**
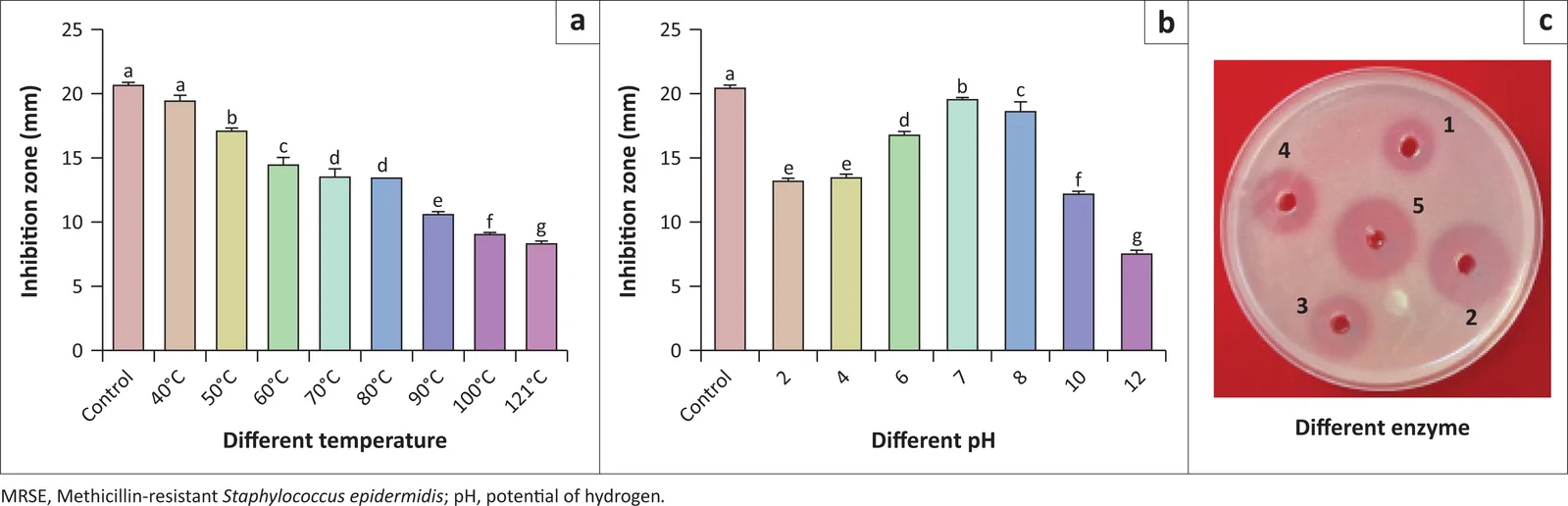
Effect of temperature, pH, and enzymatic treatment on the antibacterial activity of NELs-50. against methicillin-resistant *Staphylococcus epidermidis*. (a) Antibacterial activity of NELs-50 after exposure to different temperatures, showing a gradual decrease in inhibition zone with increasing temperature; (b) Antibacterial activity of NELs-50 at various pH levels, indicating maximum activity near neutral pH.; (c) Effect of different enzymes on the antibacterial activity of NELs-50, showing variation in inhibition zones depending on enzymatic treatment (1: pepsin, 2: amylase, 3: proteinase K, 4: trypsin, 5: untreated) (01 September 2024 to 30 June 2025, Hainan, China).

### Minimum inhibitory concentration determination

The MIC was determined by broth microdilution assays. Serial dilutions of the lyophilised ethyl acetate extract were prepared and tested against the target bacterial pathogen in 96-well microplates. Following 24-h incubation at 37 °C, the MIC of the ethyl acetate extract from strain NELs-50 was determined to be 125 µg/mL, representing the lowest concentration that completely inhibited visible bacterial growth.

### Gas chromatography-mass spectometry analysis of the bioactive extract

Analysis by GC-MS revealed 11 chromatographic signals; after excluding duplicate peaks and one non-identified compound, nine putative metabolites were considered as potential contributors to the observed anti-MRSE activity, including ionic compounds, heterocyclic compounds, amides, phenols, alcohols, and esters. These compounds are detailed in Table 4, with their retention times, chemical names, compound types, and peak area percentages. The GC-MS analysis revealed pyrrolo[1,2-a]pyrazine derivative as the most prominent compounds with peak areas of 100%, followed by ammonium acetate at 98.83% peak area. Other significant compounds included 2,4-Di-tert-butylphenol (12.64%), benzeneacetamide (9.35%), and various fatty acid esters, representing diverse chemical classes with potential antimicrobial properties.

**TABLE 4:** Compounds identified from NELs-50 extract using gas chromatography-mass spectometry (01 September 2024 to 30 June 2025, Hainan, China).

No.	Retention time	Compound	Type	Area (%)
1	3.610	Ammonium acetate	Ionic compound	98.83
2	14.826	5-Thiazoleethanol, 4-methyl-	Heterocyclic compound	6.80
3	18.899	Benzeneacetamide	Amide	9.35
4	21.575	2,4-Di-tert-butylphenol	Phenol	12.64
5	22.930	Caryophyllenyl alcohol	Alcohol	4.24
6	23.550	Not determined	Not determined	10.74
7	29.701	Hexadecanoic acid, methyl ester	Ester (fatty acid ester)	5.88
8	32.015	9-Octadecenoic acid, methyl ester, (E)-	Ester (fatty acid ester)	3.19
9	38.334	Pyrrolo[1,2-a]pyrazine-1,4-dione, hexahydro-3-(phenylmethyl)-	Heterocyclic compound	100.00
10	38.835	Pyrrolo[1,2-a]pyrazine-1,4-dione, hexahydro-3-(phenylmethyl)-	Heterocyclic compound	55.58
11	44.512	13-Docosenamide, (Z)-	Amide (fatty amide)	9.08

No., number.

## Discussion

The isolation of *S. pseudogriseolus* NELs-50 from Hainan mangroves demonstrates the promising antimicrobial potential of mangrove-derived actinomycetes. While terrestrial *Streptomyces* have been extensively exploited for antibiotic production, mangrove-associated strains remain largely unexplored^26^ despite exposure to unique selective pressures that drive novel biosynthetic capabilities.^27,28^ Extreme mangrove conditions likely contribute to specialised secondary metabolite pathways absent in terrestrial counterparts, as demonstrated by the potent antimicrobial activity of mangrove-derived strain NELs-50.^29,30,31^ These unique environmental pressures select for microorganisms with enhanced bioactive compound production capabilities, explaining the strong anti-MRSE activity observed in this study.^31^ The optimisation findings demonstrate the importance of systematic parameter evaluation for maximising antimicrobial production from mangrove-derived *Streptomyces*. Although the laboratory-optimised conditions may not fully replicate the complex mangrove environment, they provide a practical basis for improving antimicrobial metabolite production during future bioprocess development. The stability assays showed that the ethyl acetate extract retained activity across a broad pH range but was sensitive to elevated temperatures, with only 41% residual activity after treatment at 121 °C. Therefore, the extract should not be described as thermostable; rather, it exhibits partial thermal stability under moderate heat exposure. In addition, its resistance to amylase and partial sensitivity to proteolytic enzymes suggest that the active substances are likely non-proteinaceous or contain non-protein components, which is consistent with many secondary metabolites produced by *Streptomyces*. These properties may still offer practical advantages for extraction, handling, and preliminary pharmaceutical development, although further purification and compound-specific stability testing are required. The observed MIC of 125 µg/mL demonstrates moderate anti-MRSE potency, though further evaluation against diverse clinical isolates and toxicity profiling will be necessary to assess therapeutic viability.

The chemical diversity revealed by GC-MS analysis presents both opportunities and challenges for therapeutic development. Most notably, pyrrolo[1,2-a]pyrazine derivative dominates the profile with notable peak areas of 100%, representing the most abundant constituents. These heterocyclic scaffolds are particularly significant as they represent underexploited chemical space with demonstrated antimicrobial activity against various pathogens.^32,33^ The dominance of ammonium acetate (98.83% peak area) likely reflects extraction artefacts rather than genuine bioactive content and should not be considered among the bioactive compounds; this observation emphasises the need for refined purification protocols to eliminate such contaminants. Excluding this artefact, the presence of 2,4-Di-tert-butylphenol (12.64%), a known antimicrobial agent,^34^ alongside various fatty acid esters and the pyrrolo[1,2-a]pyrazine derivative suggest multiple bioactive mechanisms contributing to overall anti-MRSE activity.

### Limitations

Several limitations constrain the broader impact of these findings. Evaluation against only a single MRSE strain limits generalisability across clinical variants, while undefined mechanisms of action hinder rational optimisation strategies. Future investigations should prioritise bioassay-guided fractionation to isolate individual compounds, particularly the dominant pyrrolo[1,2-a]pyrazine derivatives, followed by comprehensive structure-activity relationship studies and mechanistic investigations. Broader antimicrobial profiling against panels of drug-resistant pathogens would establish therapeutic spectrum and guide clinical applications.

## Conclusion

The anti-MRSE activity of *S. pseudogriseolus* NELs-50 highlights the potential of mangrove ecosystems as reservoirs of bioactive secondary metabolites. This study contributes to the growing body of evidence supporting marine-derived actinomycetes as valuable resources for combating antimicrobial resistance.
